# COVID-19 pandemic decreased sleep quality of medical students

**DOI:** 10.5935/1984-0063.20220075

**Published:** 2022

**Authors:** Ana Clara Aragão Fernandes, Deborah de Melo Magalhães Padilha, Anaís Concepción Marinho Andrade de Moura, Carlos Eduardo França de Aquino, Irinna Bruna de Araújo Lima, Sergio Arthuro Mota-Rolim

**Affiliations:** 1 Potiguar University, Medical School - Natal - Rio Grande do Norte - Brazil; 2 Potiguar University, Department of Statistics - Natal - Rio Grande do Norte - Brazil; 3 Federal University of Rio Grande do Norte, Brain Institute, Department of Physiology and Behavior and Onofre Lopes University Hospital - Natal - Rio Grande do Norte - Brazil

**Keywords:** COVID-19, Sleep, Medical Students, Sleep Quality, PSQI

## Abstract

**Objective:**

Medical students are especially vulnerable to situations of poor sleep quality due to academic demands. The COVID-19 pandemic brought significant changes and high psychological stress, causing a great impact on this population. Here we aim to analyze the influence of the pandemic on the sleep quality of medical students.

**Methods:**

Cross-sectional, observational, and descriptive study with a quantitative approach carried out with students from medical universities in Rio Grande do Norte state (Brazil) through the online application of two questionnaires: Pittsburgh sleep quality index (PSQI-BR) and sociodemographic questionnaire (SQ).

**Results:**

A total of 142 medical students participated in this study: 103 women and 39 men. We observed a prevalence of low sleep quality in 78.16% of the sample and that the pandemic significantly affected the sleep quality among medical students (*p*<0.05). We also found an alteration in the sleep pattern in 83% of the participants, mainly due to anxiety symptoms (38%). Finally, we observed no statistically significant difference in sleep quality or sleep patterns between genders or college period (*p*>0.05).

**Discussion:**

This rate of poor sleep quality is higher than the prevalence of periods before the pandemic (58%). Concerns about COVID-19’s negative impact on medical education, delayed training, and impact on the generation of medical jobs can directly aggravate the sleep quality.

**Conclusion:**

The COVID-19 pandemic negatively influenced the sleep quality of medical students, increasing the prevalence of poor sleep quality and promoting changes in the sleep pattern.

## INTRODUCTION

Coronavirus disease 2019 (COVID-19) is a highly contagious viral illness caused by severe acute respiratory syndrome coronavirus 2 (SARS-CoV-2), which caused a catastrophic effect on the world’s health. The first cases of this predominantly respiratory viral illness were first reported in Wuhan, Hubei Province, China, in late December 2019. SARS-CoV-2 rapidly disseminated across the world in a short span of time, compelling the World Health Organization (WHO) to declare it as a global pandemic on March 11, 2020. The pandemic has also resulted in the loss of livelihoods due to prolonged shutdowns, which have had a rippling effect on the global economy.

The pandemic also impacted negatively people’s sleep across the world^1^. Besides this general loss of sleep quality, medical students are a subgroup that is especially vulnerable to situations of poor sleep quality due to the intensity of studies and extensive workload, in addition to situations such as night shifts, frequent evaluation exams, and full class schedules^2^. Along with this, situations that impair their quality and quantity of sleep are the environmental and social stress they are subjected to in their workplace^3,4^. Furthermore, regarding academic performance, a study by Medeiros et al. (2001)^5^ points to the presence of circadian sleep irregularity in this group, which is also associated with deprivation and low quality of sleep. This implies an increase in stress, which influences negatively learning and academic performance.

During the COVID-19 pandemic, young adults reported experiencing symptoms of anxiety, depression, and a high level of stress, which could directly influence sleep quality^6-8^. Another study carried out during this period confirmed the high prevalence of low quality of sleep, sleep-wake disorders, and use of hypnotics in college students compared to high school students^9^. In addition to this chronic scenario of poor sleep quality, the quarantine during the COVID-19 pandemic brought traumatic events and high psychological stress such as social isolation, death of family members, fear of infection, suspension of theoretical and practical classes, adoption of online platforms, and fears about medical careers^10^.

To date, as far as we know, there is no study that addresses the impact of the COVID-19 pandemic on the sleep of Brazilian medical students. Thus, it is urgent to understand the influence of this period on quality of sleep in order to adopt public health measures that aim to improve the quality of life and sleep of this population at risk. The present study intends to analyze the influence of the pandemic on the quality of sleep of medical students.

## MATERIAL AND METHODS

This is a cross-sectional, observational and descriptive study with a quantitative approach carried out in a virtual environment targeting higher education medical institutions in Rio Grande do Norte through the online application of two questionnaires: Pittsburgh sleep quality index (PSQI-BR) and sociodemographic questionnaire (QSD) - from January to March 2021.

The participants were medical students duly enrolled in higher education institutions in Rio Grande do Norte, from the 1^st^ to the 6^th^ year and over 18 years of age. Exclusion criteria were: students with any psychiatric diagnosis - factor questioned when filling out the questionnaire and, if so, excluded from the results.

For data collection, the Academic and Athletic Centers of each institution were contacted to identify the student-leaders of each class, who were responsible for sending Google Forms via email and WhatsApp containing the PSQI-BR and the QSD. Initially, the informed consent was presented, containing the interests, risks and benefits of the study, the destination of the data, making clear the voluntary and free participation of the participant. After signing the consent form, they were asked to fill in the QSD questionnaires with information about age, gender, academic year, location of graduation, professional performance, presence of psychiatric diagnosis and use of stimulant substances and alcohol, and finally, change in sleep pattern during the quarantine period.

Finally, they were asked to complete the PSQI-BR questionnaire, which assesses sleep quality over a period of 1 month. The questionnaire consists of 19 self-assessments and 5 questions that must be answered by roommates or people who sleep in the same bed as the participant. The 19 questions are categorized into 7 components, graded in scores that range from 0-3. The components are: subjective sleep quality (C1), sleep latency (C2), sleep duration (C3), current sleep efficiency (C4), sleep disturbances (C5), use of sleep-inducing medication (C6), and daytime dysfunction (C7). The sum of the scores of the 7 components leads to a global score, which ranges from 0-21. An overall PSQI score >5 indicates poor sleep quality in at least 2 components or moderate difficulty in more than 3 components^11^.

The data obtained from the research were organized in an Excel spreadsheet and statistically analyzed by SPSS version 12. A frequency distribution table of the variables tested was built. Differences were considered significant when *p*<0.05 using chi-square or Fisher’s exact tests.

The participants of this study were clarified about the research objectives, presented the consent form and later, only for those who agreed to participate in the research, the questionnaires were presented. The research was submitted to the research ethics committee of Potiguar University and approved (Opinion No. 4,455,154), observing all the ethical aspects recommended in research with human beings.

## RESULTS

Table 1 shows the description of our sample, with numbers by gender, age, year of graduation, location, and profession. In summary, 142 medical students participated in this study: 103 women (mean age 22.01 +/- 3.3 years old) and 39 men (mean age 22.35 +/- 3.69 years old).

**Table 1:** Demographics characteristics of the study sample (n=142).

Variable	n (%)
**Gender**	
**Female**	103 (72.02%)
**Male**	39 (27.27%)
**Age**	
**18-21**	48 (33.56%)
**21-24**	64 (44.75%)
**24-27**	18 (12.58%)
**>27**	11 (7.6%)
**Year of graduation**	
**1^st^ year**	33 (23.07%)
**2^nd^ year**	29 (20.27%)
**3^rd^ year**	35 (24.47%)
**4^th^ year**	33 (23.07%)
**5^th^ year**	11 (7.69%)
**6^th^ year**	01 (0.69%)
**Location**	
**Natal**	94 (65.73%)
**Mossoró**	42 (29.37%)
**Caicó**	06 (4.19%)
**Ocupation**	
**Student**	137 (95.80%)
**Worker**	05 (3.49%)

The prevalence of sleep quality in medical students stratified by sex and period is shown in Tables 2 and 3. The high prevalence of low sleep quality in the studied group is observed, being present in 78.16% (n=111) of the total sample. It is important to emphasize that there was no statistically significant difference (*p*>0.05) in sleep quality between genders and college periods.

**Table 2:** Prevalence of sleep quality in medical students stratified by sex (n=142).

Variable	Male N=39 (27.27%)	Female N=103 (72.02%)	x^2^	p-value
	n (%)	n (%)		
**Sleep quality**			0,0264	0,87
**Poor**	31 (78.90%)	80 (77.7%)		
**Good**	08 (21.1 %)	23 (22.3 %)		

Note: Low quality of sleep was considered a global PSQI score >5.

**Table 3:** Prevalence of sleep quality in medical students stratified by graduation year (n=142).

Variable	First year N=33 (23.07%)	Second year N=29 (20.27%)	Third year N=35 (24.47%)	Fourth year N=33 (23.07%)	Fifth year N=11 (7.69%)	Sixth year N=1 (0.69%)	x^2^	p-value
	n (%)	n (%)	n (%)	n (%)	n (%)	n (%)		
Sleep quality							0.21	0.13
Poor	27 (81.8%)	22 (75%)	28 (79.0%)	22 (65.6%)	10 (90%)	1 (100%)		
Good	06 (18.2%)	07 (25%)	07 (20.1%)	11 (34.4%)	01 (10%)	0		

Note: Low quality of sleep was considered PSQI score >5.

Regarding the characteristics of sleep alteration during the COVID-19 pandemic (Table 4), an alteration in the sleep pattern was observed in 83% of the participants. Of those who claimed to have an alteration in sleep, 47% claimed to be an alteration for more hours of sleep and 53% declared an alteration for less sleep. When asked about the reasons for changing this pattern, 38% said they had anxiety symptoms and 15% said they had a large number of activities to be done.

**Table 4:** Characteristics of poor sleep quality during the pandemic (COVID-19) among medical students in the state of Rio Grande do Norte-RN.

Parameters evaluated	Medical students of Natal City - RN during the pandemic		
Sleep pattern change	YES	NO	
	83%^a^	17%^b^	
	For more	For less	
	47%^a^	53%^a^	
Gender	Male	Female	
	42%^a^	58%^a^	
Reason for sleep disturbance	Anxiety symptoms	Many activities	Other^*^
	38%	15%	47%

Notes:

a,b Different letters on the same line differ;

* Other causes: On cell phones, watching TV, insomnia, among others.

According to data analysis, it was found that the pandemic affected the quality of sleep among medical students (*p*<0.05), however when comparing the sexes there was no statistical difference (*p*>0.05). Furthermore, regarding the type of change (more hours or fewer hours), there was also no statistical difference (*p*>0.05). The most frequently cited reason for the change was anxiety (38%) (Table 4).

## DISCUSSION

The present study shows a high prevalence of poor sleep quality among medical students during the COVID-19 pandemic, with an alteration in their sleep pattern due to symptoms of anxiety and many activities in college. For these analyzed variables, no difference between genders or graduation period was observed.

The Pittsburgh sleep quality questionnaire (PSQI) revealed a prevalence of 78.16% of participants with a PSQI-BR score >5, indicating the presence of poor sleep quality. This rate is higher than that found in other studies carried out during a pandemic with medical students in China (33.2%)^10^ and nursing students in Spain (60.4%)^12^. In addition, assessing only medical undergraduates, the prevalence is also higher when compared to periods before the pandemic, with this figure ranging from 51.5%^13^, 58%^4^, and 64%^14^. This demonstrates a prevalence of poor sleep quality in this risk group, which has worsened sleep quality even further during the COVID-19 pandemic.

In this scenario, a Chinese study observed lower sleep quality in fifth-year medical students, a fact suggested due to factors such as pressure for residency and employability tests after this pandemic period^10^. In addition, this work also observed a greater prevalence of this disorder in females, which can be explained by the way in which emotional stress situations prevail^10,15^. This demonstrates a prevalence of poor sleep quality in this risk group, which has worsened during the COVID-19 pandemic.

Medical students are a vulnerable group to situations of low quality of sleep due to the intensity of activities and workload^4^. Moreover, concerns about COVID-19’s negative impact on medical education, delayed training, and impact on the generation of medical jobs can directly aggravate the sleep quality of this group^10^. During the pandemic in Brazil, there was a discrepancy between public and private faculties of medicine, in which the latter remained activities in the online format, while the first one came to a complete standstill. Unfortunately, this situation did not provide solutions for the neediest groups, which did not have adequate technologies for viewing online classes. We believe that this situation could be another reason for the increased concern and stress.

Home confinement drastically changed daily activities and, consequently, sleep patterns. It was observed in university students higher sleep latency, increased time in bed and later times to wake up; however, paradoxically, they also had poor sleep quality^7,9,12^. According to Cellini et al. (2020)^7^, in a study carried out with 809 university students, confinement at home was marked by a prevalence of 32.6% of moderate to severe anxiety symptoms, with changes in the sleep pattern and high use of electronics at night. This fact was also observed by Romero-Blanco et al. (2020)^12^ when analyzing nursing students, in which sleep alterations were related to the group with reports of anxiety and depression.

Corroborating with the literature, in our study we observed alterations in the sleep pattern, but there was no significant difference between the duration of sleep for more or fewer hours. Furthermore, the main reason related to this change is the presence of anxiety in 38% of the participants and, secondarily, the presence of too many activities to be performed in 15% of them. This last fact deserves to be highlighted, since no study to date has mentioned the overload of university activities during the pandemic, as it was theorized that due to social isolation and suspension of the demands of face-to-face medical training, online activities would require less effort and would not impact negatively on the quality of life of students.

Depression and other mental illnesses are frequent among medical students. Anxiety is the most prevalent disorder, with studies showing an index of 89.6% in this group^13^. In conjunction with this, social isolation and confinement at home are risk factors for mental illnesses as it reduces contact with family and friends^9^, as well as the practice of physical activities and sun exposure^7^, consequently increasing the level of stress. Another important point about home confinement is the increased use of digital devices before bedtime, which affects latency and sleep pattern^7^, a fact also observed in our work. All these factors in a previously prone group contribute to the worsening of mental health and also to the worsening of sleep quality, both coefficients are also observed here.

It is important to highlight that poor sleep quality and mental disorders are related to a low immune response, thus these risk groups may be more prone to infection by SARS-CoV-2 or other pathogens^16^. In this way, intervention to prevent or treat these conditions in medical students is extremely important for public health. Aiming at improving this scenario, investment in actions to encourage self-care is necessary to act efficiently in this risk group, as educational actions on sleep medicine, such as the addition of a mandatory module in the medical curriculum, can increase knowledge in this area. However, this activity is not related to an increase in the quality of sleep in this group^17^. It is suggested that medical students already have some knowledge about the benefits of performing sleep hygiene and the importance of its quality, but they choose not to prioritize sleep health due to the demands of medical training or the lifestyle itself^4^.

Our study has several limitations. First, this is a cross-sectional observational study and is not suitable for conducting cause-and-effect relationships. Second, it was carried out with a non-probabilistic sample with volunteer participants and only from Rio Grande do Norte, which can result in selection bias. Studies with a larger sample and with collections in several Brazilian states must be done. Third, because this study is carried out with data collection through an online questionnaire that participants fill out on their own, they may present discrepant data from the clinical evaluation by medical professionals. Another noteworthy point is that the majority of the sample was female, which is more likely to develop anxiety symptoms and sleep disorders^10,17^, thus this fact may have significantly contributed to the results. Future studies should carry out the sampling aiming at equity in quantity between the sexes.

To conclude, we observed that the COVID-19 pandemic negatively influenced the sleep quality of medical students, increasing the prevalence of poor sleep quality and promoting changes in the sleep pattern. In addition, a high rate of anxiety in this group was observed, which was the main reason for changes in the sleep pattern. Another fact that deserves mention is the presence of overload of university activities during this period of confinement, which also negatively influenced sleep. The presence of poor sleep quality is a public health issue and this study highlights the importance of monitoring the sleep health of medical students by the educational institution both during the COVID-19 pandemic and after it, providing self-care and support interventions to help students overcome these problems and improve their quality of life. In addition, it is necessary to evaluate the institutions regarding the medical curriculum and the number of activities in this training, to avoid overload and exhaustion of students. Future research should be carried out using longitudinal monitoring of students throughout the course, aiming to identify potential aggravating factors and really effective interventions to improve the quality of sleep in this group.
